# The prM-independent packaging of pseudotyped Japanese encephalitis virus

**DOI:** 10.1186/1743-422X-6-115

**Published:** 2009-07-30

**Authors:** Hee Jung Lee, Kyung-Il Min, Jungeun Lee, Sin-Hyung Kang, Wonkyung Jeon, Jae Hwan Nam, Young Ran Ju, Young Bong Kim

**Affiliations:** 1 Department of Animal Biotechnology, College of Animal Bioscience & Technology, Konkuk University, 1 Hwayang-dong, Gwangjin-gu, Seoul 143-701, Republic of Korea; 2 Virus Vaccines Division, Korea Food & Drug Administration, 194 Tongilro, Eunpyeong-gu, Seoul 122-704, Republic of Korea; 3 Center for Herbal Medicine Improvement Research, Korea Institute of Oriental Medicine, 483 Expo-ro, Yuseong-gu, Daejeon 305-811, Republic of Korea; 4 Department of Biotechnology, The Catholic University of Korea, 43-1 Yeokgok 2-dong, Wonmi-gu, Bucheon, Gyeonggi-do 420-743, Republic of Korea; 5 Division of Arboviruses, Center for Immunology & Pathology, National Institute of Health, Korea Centers for Disease Control and Prevention, 194 Tongilro, Eunpyeong-gu, Seoul 122-701, Republic of Korea

## Abstract

As noted in other flaviviruses, the envelope (E) protein of Japanese encephalitis virus (JEV) interacts with a cellular receptor and mediates membrane fusion to allow viral entry into target cells, thus eliciting neutralizing antibody response. The formation of the flavivirus prM/E complex is followed by the cleavage of precursor membrane (prM) and membrane (M) protein by a cellular signalase. To test the effect of prM in JEV biology, we constucted JEV-MuLV pseudotyped viruses that express the prM/E protein or E only. The infectivity and titers of JEV pseudotyped viruses were examined in several cell lines. We also analyzed the neutralizing capacities with anti-JEV sera from JEV-immunized mice. Even though prM is crucial for multiple stages of JEV biology, the JEV-pseudotyped viruses produced with prM/E or with E only showed similar infectivity and titers in several cell lines and similar neutralizing sensitivity. These results showed that JEV-MuLV pseudotyped viruses did not require prM for production of infectious pseudotyped viruses.

## Findings

Japanese encephalitis virus (JEV) is a serious mosquito-borne flavivirus that causes pandemic infectious disease of major public health importance in Asia. JEV is a member of the genus *Flavivirus *in the family *Flaviviridae*, which includes yellow fever virus, Dengue virus, West Nile virus, and St. Louis encephalitis virus [1,2].

The JEV single-stranded RNA genome (≈ 11 kb) encodes three structural proteins – capsid (C), premembrane (prM) or membrane (M), and envelope (E) protein – and seven nonstructural (NS1, NS2A, NS2B, NS3, NS4A, NS4B, and NS5) proteins [3-5].

The assembly of JEV in the endoplasmic reticulum is followed by modification of the two envelope proteins E and prM and virion export through the secretory pathway. PrM (≈ 26 kDa) is a precursor of the membrane-anchored and it cleaved a soluble Pr peptide and virion associated M protein (≈ 8 kDa) by trans-Golgi resident furin or related enzyme [6], resulting in two different forms of virion: the intracellular E- and prM-containing form, and the extracellular E- and M-containing form [3,7].

The E protein plays a major role in virus assembly, adhesion, receptor binding and membrane fusion, hemagglutination inhibition (HI), and induction of neutralizing antibodies (Nabs) [8-10]. Therefore, the E protein is the principal target of neutralization by specific antibodies against JEV infection [4,11]. E proteins of JEV expressed in different viral vector systems such as vaccinia virus, sindbis virus, and baculovirus have elicited high levels of neutralizing antibodies against JEV infection and have been tested as second generation JEV vaccines in mice [7,12-14]. From these reports, it is unknown whether prM cleavage affects infectivity, E protein expression, or induction of neutralizing activity. A major function of prM was studied by blocking prM cleavage or by mutation of the conserved glycosylation motif of JEV prM [15]. Even though the direct role of prM during the viral replication was not elucidated [6], it has a crucial function in multiple stages of JEV biology.

**Figure 1:**
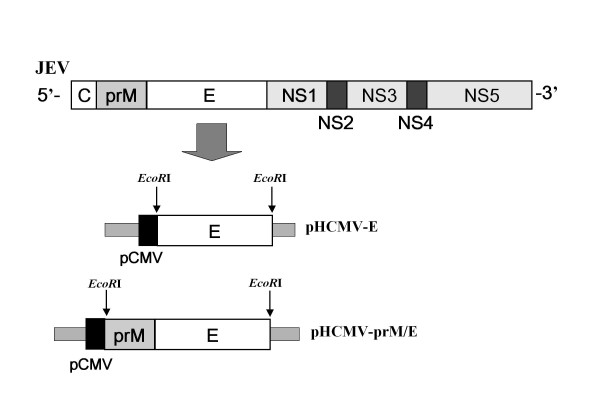
**Construction of JEV pHCMV-prM/E and pHCMV-E**. Four plasmids were produced: pHCMV-prM/E (NK or BJ) and pHCMV-E (NK or BJ).

We generated pseudotyped viruses containing the prM/E or E protein of the current JEV vaccine strains Nakayama-NIH (NK) and Beijing-1 (BJ). The DNA fragments encoding the E and prM/E regions were amplified by polymerase chain reaction (PCR) from the cDNA of the NK strain and BJ strain kindly supplied from the Department of Vaccine, KFDA, Korea. PCRs were performed using one of the two forward primers: for prM/E amplification, 5'-AATGA**GAATTC**GACCATGTGGCTCGCAAGCTTGGC-3'; and for E amplification, 5'-GGTCGC**GAATTC**TGCAGGTTCAACTGTCTGGGAGTG-3'. The reverse primer for both amplifications was 5'-ACCAATGTGCATGCTTAGCTCGA**GAATTC**CATTG-3'. Each primer had an *EcoR*I restriction site. To generate pHCMV-prM/E and pHCMV-E, the PCR products of prM/E and E were digested with *EcoR*I and subsequently cloned into pHCMV-G [16] digested with *EcoR*I (Figure 1).

Pseudotyped viruses encoding prM/E or E of JEV NK and BJ strain were produced as previously described [17]. Briefly, TELCeB6 cells, a MuLV packaging cell line [18], were transfected with pHCMV-E or pHCMV-prM/E by a calcium phosphate method. After overnight incubation, the culture medium was replaced, and the cells were incubated for two additional days. The supernatants containing pseudotyped viruses were harvested by low speed centrifugation (1,500 × *g*, 5 min) to remove cell debris.

**Figure 2:**
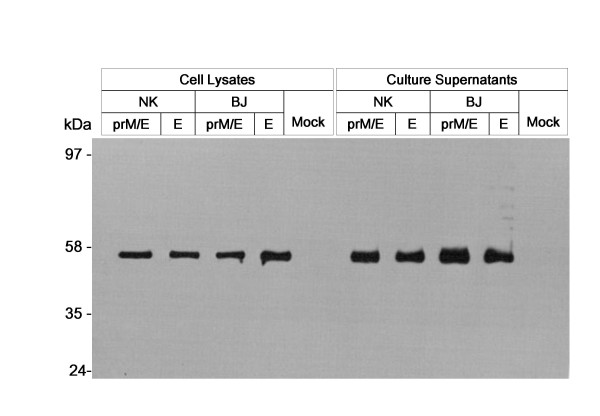
**Expression of JEV envelope (E) protein**. Cell lysate and culture media of JEV-pseudotyped viruses were subjected to SDS-PAGE. JEV-E proteins were detected by western blotting using sera from mice immunized with JEV (Nakayama-NIH strain). The bands show the JEV-E protein (53 kDa) in cell lysates and culture supernatants. Non-transfected TELCeB6 cells were used as a negative control.

Figure 2 shows the expression of the E proteins from each pseudotyped JEV constructs by western blot analysis using anti-JEV (Nakayama) sera. The envelope genes of the NK and BK strains were expressed well in cell culture supernatants and lysates. Similar amounts of E proteins were expressed in NK and BJ transfected cells.

**Figure 3:**
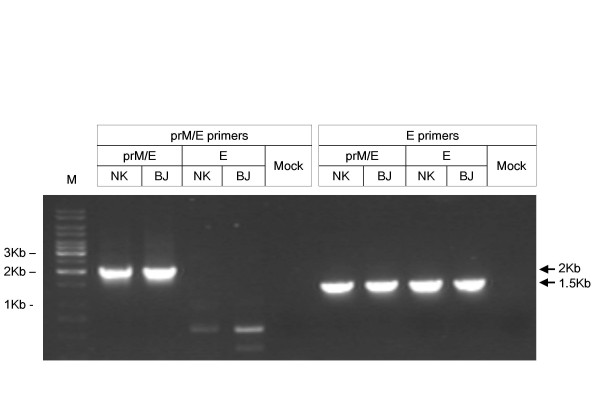
**Expression of JEV *prM *and *Env *genes in TELCeB6 cells**. TELCeB6 cells were transfected with pHCMV-prM/E and pHCMV-E of each of NK and BJ strains. RT-PCR products of the indicated size were amplified from total RNA samples extracted from TELCeB6 cells. The products correspond to 2 Kb of *prM *(amplified by prM/E primer) and 1.5 Kb of *Env *(amplified by E primer). The molecular marker (Kb) is shown on the left.

The JEV-pseudotyped viruses expressing prM-E and E were properly processed and released into the culture media with similar levels of expression. However prM-E polyprotein bands were not detected despite many attempts. To check the expression of the *prM/Env *gene during pseudotyped virus formation, we performed reverse transcription (RT)-PCR. Figure 3 shows the amplified JEV *prM/Env *(2062 bp), *Env *(1501 bp) transcripts in transfected TELCeB6 cells. We assumed that the cleavage of the prM protein is too rapid to detect with prM/E polyprotein and our sera from JEV-immunized mice did not contain the prM antibodies.

To test the effect of prM on pseudotyped viral infectivity and viral titer, infection tests with the pseudotyped viruses were carried out in in 96-well plates with Vero, PK15, CRFK, NIH3T3, HeLa, 293T, BHK-21, MDCK, and HOS cells. Four kinds of pseudotyped JEVs were added to the host cells and incubated at 37°C in a 5% CO_2 _incubator for 36 hours. All infections were done in triplicate. After X-gal staining, JEV pseudotyped virus-infected cells appeared blue. This resulted from integration of the MuLV pseudovirus genome encoding β-galactosidase. These cells were counted as an infectious unit. All JEV pseudotyped viruses could infect PK15, CRFK, NIH3T3, HeLa, 293T, BHK-21, Vero, and MDCK cells, but not HOS cells (Table 1).

**Table 1:** Infectivity and titer of JEV pseudotyped viruses in host cells

Infectious units/ml (IFU/ml)								
	Host cell							
	
JEV pseudotyed virus	Vero	BHK-21	HeLa	CRFK	PK15	293T	MDBK	HOS

NK prM/E	4.09 *10^4^	7.46 *10^3^	4.08 *10^3^	9.74 *10^4^	1.33 *10^3^	1.14 *10^2^	2.08 *10^2^	0
NK E	4.25 *10^4^	7.58 *10^3^	4.12 *10^3^	1.20 *10^5^	1.09 *10^3^	1.32 *10^2^	2.11 *10^2^	0
BJ prM/E	4.12 *10^4^	7.76 *10^3^	4.35 *10^3^	1.02 *10^5^	1.82 *10^3^	2.04 *10^2^	2.08 *10^2^	0
BJ E	4.83*10^4^	7.91 *10^3^	4.29 *10^3^	1.36*10^5^	2.90 *10^3^	2.05 *10^2^	2.23 *10^2^	0

The titers of pseudotyped viruses were comparable to the infectivity of JEV in each host cell line. JEV pseudotyped virus with E or prM/E (both NK and BJ strain) could efficiently infect several cell lines, with typical titers between 1.14 × 10^2 ^and 1.36 × 10^5 ^infectious units (IFU)/mL. From Table 1, viruses pseudotyped with prM/E exhibited infectivity similar to those pseudotyped with E only. This showed that the infectivity and titer of JEV pseudotyped virus is not affected by prM deletion.

Neutralizing sensitivity was tested with JEV-immunized immune sera, which was supplied by the Catholic University of Korea, to check the effect of prM on pseudotyped JEV antigens. As previously described [17], neutralization assays were carried out with Vero cells in triplicate. Approximately 100 IFU/mL of pseudotyped viruses were incubated with 10-fold diluted sera from mice immunized with JEV for 1 h at 37°C, and the mixture was subsequently added to Vero cells. After 2 days of incubation, virus infection was monitored by X-gal staining as described above. The neutralizing sensitivity was expressed as virus reduction by neutralizing antibodies.

**Figure 4:**
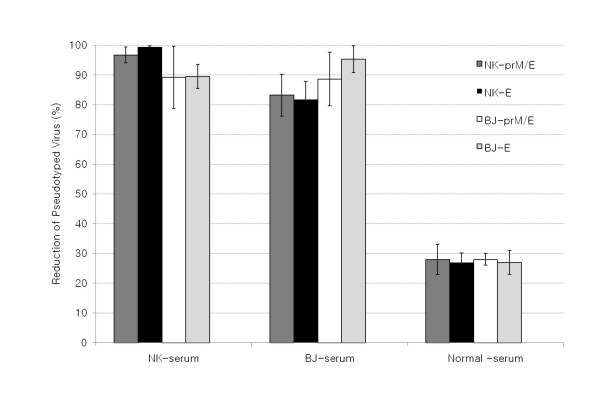
**Neutralizing assay with JEV pseudotyped viruses**. Four different JEV-pseudotyped viruses (NK-prM/E, dark grey; NK-E, black; BJ-prM/E, white; BJ-E, light grey) were incubated with sera from mice immunized with JEV-NK strain or JEV-BJ strain. Neutralizations were determined with 1:10 diluted serum.

Figure 4 shows the reduction of pseudotyped JEVs by neutralization with homologous and heterologous sera. A high neutralization was observed with 1:10 diluted serum. In contrast, no neutralizing activity was detected in the normal mouse serum (reduction was less than 30%). The neutralization by homologous sera was more complete than that by heterologous sera. However, there was no significant difference between pseudotyped JEV prM/Env and Env.

In summary, we generated pseudotyped JEVs that express prM/E or E proteins from two JEV vaccine strains NK and BJ. All four JEV pseudotyped viruses efficiently infected several cell lines and were neutralized by sera from JEV-immunized mice. The main purpose of generating JEV pseudotyped virus was to devise a safe and rapid assay system to assess neutralizing antibodies by avoiding the use of infectious, replication-competent JEVs in a Biosafety Level 3 laboratory. The titer of the four JEV pseudotyped viruses was greater than 10^4 ^IFU/ml. This confirmed the possibility of mass-producing viruses to conduct neutralization assays with Vero and CRFK cells. Even though a crucial function of prM in assembly and maturation of flaviviruses has been reported, the two types of JEV-MuLV pseudotyped virus that respectively express the E or prM/E proteins were found to have no significant difference in the level of transcription and the extent of protein expression, infectivity, titer, and neutralization sensitivity.

## Competing interests

The authors declare that they have no competing interests.

## Authors' contributions

HJL, JW, YRJ, YBK participated in the design of the study, HJL, JL, SHK performed the experiments, KIM and JHN provided key reagents, and HJL and YBK edited and approved the final manuscript.

## References

[B1] Kuno G Chang GJ Tsuchiya KR Karabatsos N Cropp CB Phylogeny of the genus Flavivirus J Virol 1998 72 73 83 9420202 10.1128/jvi.72.1.73-83.1998 PMC109351

[B2] Venugopal K Gould EA Towards a new generation of flavivirus vaccines Vaccine 1994 12 966 975 10.1016/0264-410X(94)90329-8 7975848

[B3] Yasuda A Kimura-Kuroda J Ogimoto M Miyamoto M Sata T Sato T Takamura C Kurata T Kojima A Yasui K Induction of protective immunity in animals vaccinated with recombinant vaccinia viruses that express PreM and E glycoproteins of Japanese encephalitis virus J Virol 1990 64 2788 2795 2159544 10.1128/jvi.64.6.2788-2795.1990 PMC249459

[B4] Kaur R Vrati S Development of a recombinant vaccine against Japanese encephalitis J Neurovirol 2003 9 421 431 10.1080/713831600 12907387

[B5] Wu SC Lian WC Hsu LC Liau MY Japanese encephalitis virus antigenic variants with characteristic differences in neutralization resistance and mouse virulence Virus Res 1997 51 173 181 10.1016/S0168-1702(97)00098-1 9498615

[B6] Keelapang P Sriburi R Supasa S Panyadee N Songjaeng A Jairungsri A Puttikhunt C Kasinrerk W Malasit P Sittisombut N Alterations of pr-M cleavage and virus export in pr-M junction chimeric dengue viruses J Virol 2004 78 2367 2381 10.1128/JVI.78.5.2367-2381.2004 14963133 PMC369205

[B7] Konishi E Fujii A Mason PW Generation and characterization of a mammalian cell line continuously expressing Japanese encephalitis virus subviral particles J Virol 2001 75 2204 2212 10.1128/JVI.75.5.2204-2212.2001 11160724 PMC114804

[B8] Chen HW Pan CH Liau MY Jou R Tsai CJ Wu HJ Lin YL Tao MH Screening of protective antigens of Japanese encephalitis virus by DNA immunization: a comparative study with conventional viral vaccines J Virol 1999 73 10137 10145 10559329 10.1128/jvi.73.12.10137-10145.1999 PMC113066

[B9] Kurane I Immune responses to Japanese encephalitis virus Curr Top Microbiol Immunol 2002 267 91 103 12083002 10.1007/978-3-642-59403-8_5

[B10] Mason PW Dalrymple JM Gentry MK McCown JM Hoke CH Burke DS Fournier MJ Mason TL Molecular characterization of a neutralizing domain of the Japanese encephalitis virus structural glycoprotein J Gen Virol 1989 70 Pt 8 2037 2049 10.1099/0022-1317-70-8-2037 2549181

[B11] Kojima A Yasuda A Asanuma H Ishikawa T Takamizawa A Yasui K Kurata T Stable high-producer cell clone expressing virus-like particles of the Japanese encephalitis virus e protein for a second-generation subunit vaccine J Virol 2003 77 8745 8755 10.1128/JVI.77.16.8745-8755.2003 12885894 PMC167253

[B12] Wu HH Chen CT Lin YL Lee ST Sub-fragments of the envelope gene are highly protective against the Japanese encephalitis virus lethal infection in DNA priming – protein boosting immunization strategies Vaccine 2004 22 793 800 14741175 10.1016/j.vaccine.2003.02.001

[B13] Zhao Z Wakita T Yasui K Inoculation of plasmids encoding Japanese encephalitis virus PrM-E proteins with colloidal gold elicits a protective immune response in BALB/c mice J Virol 2003 77 4248 4260 10.1128/JVI.77.7.4248-4260.2003 12634382 PMC150624

[B14] Konishi E Pincus S Paoletti E Shope RE Burrage T Mason PW Mice immunized with a subviral particle containing the Japanese encephalitis virus prM/M and E proteins are protected from lethal JEV infection Virology 1992 188 714 720 10.1016/0042-6822(92)90526-U 1585642

[B15] Kim JM Yun SI Song BH Hahn YS Lee CH Oh HW Lee YM A single N-linked glycosylation site in the Japanese encephalitis virus prM protein is critical for cell type-specific prM protein biogenesis, virus particle release, and pathogenicity in mice J Virol 2008 82 7846 7862 10.1128/JVI.00789-08 18524814 PMC2519568

[B16] Burns JC Friedmann T Driever W Burrascano M Yee JK Vesicular stomatitis virus G glycoprotein pseudotyped retroviral vectors: concentration to very high titer and efficient gene transfer into mammalian and nonmammalian cells Proc Natl Acad Sci USA 1993 90 8033 8037 10.1073/pnas.90.17.8033 8396259 PMC47282

[B17] Kim YB Lee MK Han DP Cho MW Development of a safe and rapid neutralization assay using murine leukemia virus pseudotyped with HIV type 1 envelope glycoprotein lacking the cytoplasmic domain AIDS Res Hum Retroviruses 2001 17 1715 1724 10.1089/08892220152741414 11788023

[B18] Schnierle BS Stitz J Bosch V Nocken F Merget-Millitzer H Engelstadter M Kurth R Groner B Cichutek K Pseudotyping of murine leukemia virus with the envelope glycoproteins of HIV generates a retroviral vector with specificity of infection for CD4-expressing cells Proc Natl Acad Sci USA 1997 94 8640 8645 10.1073/pnas.94.16.8640 9238030 PMC23056

